# Who is allowed to study medicine? – regulations and evidence

**DOI:** 10.3205/zma001218

**Published:** 2019-02-15

**Authors:** Wolfgang Hampe, Martina Kadmon

**Affiliations:** 1 University Hospital Hamburg-Eppendorf, Inst. of Biochemistry and Molecular Cell Biology, Hamburg, Germany; 2 University of Augsburg, Medical Faculty Augsburg, Deanery, Augsburg, Germany

## Editorial

An evidence-based selection procedure for medical student admission has a stronger impact on learning and study performance than many other educational interventions in undergraduate medical education [1]. Nevertheless, student selection internationally includes a broad range of procedures with varying evidence, ranging from selection by lottery [2] to mere selection on the ground of school leaving grades [3]. Additional student admission tests (SAT) are frequently used, since their results reliably predict study success as measured by cognitive outcome criteria [4], [5]. Complementary interview techniques on different levels of standardization are also often included as components of selection procedures [6]. While unstructured interviews do not add to the prediction of study success highly structured communication situations as in multiple mini-interviews [7], [8] predict non-cognitive study performance.

The number of applicants to undergraduate medical education programs exceeds by far the number of study places available. Even though a shortage of medical doctors is currently discussed only a limited number of additional places are offered due to the high cost of undergraduate medical training. Regulations as to who is admitted to medical education should consider both the interests of applicants concerning a fair chance in the competition for a study place as well as the public interest and an adequate and cost-efficient health care for the community. Moreover, admission decisions should be evidence-driven due to their impact on study performance [1].

After two judgments of the Federal Constitutional Court in the 1970s [9], [10] student admission to undergraduate medical training, which had so far been regulated by the individual medical schools, was standardized and unified by a treaty between the German Federal States and the German Higher Education Framework Act. Apart from the school leaving grade the waiting period after the final secondary school examination (Abitur) was considered for admission to medical school. Thus, applicants did not only get a chance for a study place, as demanded by the Federal Constitutional Court, but could even rely on a guarantee for admission to undergraduate medical training if only they had the patience to wait long enough. The detailed organization of various admission quotas, additional selection criteria and the participation of the individual medical schools in the national selection procedure have changed several times since. Following the German Federal States treaty 2006 three main quotas were introduced: 20% of the study places are allocated on the basis of the best school leaving grade only (quota for best school graduates), 20% merely on the basis of the waiting period (waiting quota) and 60% according to selection criteria of the individual medical faculties (individual medical school quota).

The predictive validity of the school leaving grade for the study success is undisputed [3]. Meanwhile, however, even the highest average school leaving grade of 1,0 requires additional luck of draw to get a study place in almost all federal states of Germany. The waiting period as selection criterion has a negative impact on study success [11], [12]. Nevertheless, applicants at the moment still wait seven to eight years for admission to undergraduate medical training. In its judgment from December 2017 the Federal Constitutional Court declared several aspects of the current national admission policy as unconstitutional, among them the length of the waiting period in the waiting quota and the selection on the basis of applicants’ preference for study place locations and the missing compensation for school leaving grades from different federal states in the individual medical school quota [13]. Admission decisions must follow the fundamental principle of aptitude for the training and the profession, and the selection process must be standardized, structured and evidence-based. Also, politics wants to develop legal regulations: In the Masterplan for Medical Training 2020 the federal government and states demand, that the selection policy should focus more on abilities that are important for the medical profession. To that end more weight should be placed on previous vocational training in health care professions, SATs and interview techniques. However, evidence for supporting vocational trainings and at least unstructured interviews as selection criteria is missing [3].

In the course of 2018, representatives of the Standing Conference of Education Ministers worked on a new German Federal States treaty for student admission [14], which will apply as of 2020. The representatives of the federal states did not agree on a fundamentally new and transparent procedure, but on regulations that may even increase the complexity of the admission process:

1. The **waiting quota** will be abolished. Since the dropout rate of students admitted via the waiting quota is considerably higher than in the other quotas [4], [11], this decision is consistent with the existing evidence. During a transition period until 2021 credits for the waiting period will still be granted in the **additional personal suitability quota** (see below), so that applicants having waited for years still get a chance for admission.
2. The **quota for best school graduates** will be increased from 20% to 30% of the study places. This decision is also supported by good evidence, since the school leaving grade is a good predictor of study success as measured by cognitive outcome parameter [3], [15].
3. In a new **additional personal suitability quota** 10% of the study places will be allocated without consideration of school grades. Eligible criteria are the results of SATs or professional experience in health care professions. The application of SATs is based on good evidence, while the evidence for the benefit of professional experience is missing [4], [8], [16].
4. **60%** of the study places will continue to be allocated according to **individual medical school criteria**. Apart from school leaving grades the individual medical school quota must consider an SAT and an additional criterion independent of school grades. Complex interview procedures are possible and grounded on evidence [6], [7], [17]. Only in case of the use of complex admission procedures the individual medical school may limit the admission to applicants choosing the respective medical school as first priority. For school leaving grades from different federal states a compensating mechanism will be introduced [18].

The admission procedures will continue to be carried out centrally by the Foundation for University Admissions (hochschulstart). The separate software solutions currently used for the study courses with national restrictions, medicine, dentistry, veterinary medicine and pharmacy and the study courses with regional restrictions will be joined. This process will require a transition phase until at least 2021, during which the medical faculties will not be able to perform complex on-site selection procedures like interview techniques. The vast majority of medical faculties will use the German Aptitude Test for Medical Studies (TMS), while a few will apply the Hamburg Assessment Test for Medicine, Natural Sciences (HAM-Nat), a situational judgment test and, for dentistry, a mental rotation test

It will not be easier in the future to apply for a place to study medicine. The guarantee to be admitted through waiting for years will be dropped. Applicants without best school leaving grades will soon have to not only compare the regulations concerning the individual medical school quota but also those of the additional personal suitability quota between the different medical faculties in order to enhance their chances for admission. One advantage is, though, that strategic considerations concerning preferences for study place locations will play a lesser role. 

The medical faculties will have to develop new concepts for their selection procedures: Which criteria should be used for selecting applicants in the individual medical school quota and in the additional personal suitability quota? And which weight should be given to those criteria? Faculties performing complex on-site selection procedures at the moment will not be able to use them during a transition phase. It remains to be seen, how many medical schools will return to complex interview techniques after this transition phase.

The newest judgment of the Federal Constitutional Court also includes specifications on the use of standardized and valid selection criteria that should predict the suitability of applicants for medical training and the profession as a doctor [13]. Since the middle of 2018 a research consortium on student selection (stav) including six consortium partners and an additional 20 cooperation partners is funded by the Federal Ministry of Education and Research. The work of this consortium is directed toward analysing various selection procedures, developing them further on the basis of data, and finally make them available to all medical faculties. In order to further develop SATs, which will play a major role in the new German Federal States treaty for student admission, a first study will include a parallel administration of different cognitive tests at several locations in spring 2019, in order to examine their discriminant and prognostic validity and possibly determine the best possible combinations of subtests.

## Competing interests

The authors declare that they have no competing interests. 
